# Cr-MOF-Based Electrochemical Sensor for the Detection of P-Nitrophenol

**DOI:** 10.3390/bios12100813

**Published:** 2022-10-01

**Authors:** Chao Hu, Ping Pan, Haiping Huang, Hongtao Liu

**Affiliations:** 1 School of Chemistry and Chemical Engineering, Jiangxi University of Science and Technology, Ganzhou 341000, China; 2 Staff Hospital of Central South University, Central South University, Changsha 410083, China; 3 College of Chemistry and Chemical Engineering, Central South University, Changsha 410083, China

**Keywords:** nanoparticle, modified electrode, electrochemical sensor, electrocatalytic activity, detection limit

## Abstract

Cr-MOF nanoparticles were synthesized by a simple hydrothermal method, and their morphology and structure were characterized by SEM, TEM, and XRD techniques. The Cr-MOF modified glassy carbon electrode (Cr-MOF/GCE) was well constructed and served as an efficient electrochemical sensor for the detection of p-nitrophenol (p-NP). It was found that the Cr-MOF nanoparticles had significant electrocatalytic activity toward the reduction of p-NP. The Cr-MOF-based electrochemical sensor exhibited a low detection limit of 0.7 μM for p-NP in a wide range of 2~500 μM and could maintain excellent detection stability in a series of interfering media. The electrochemical sensor was also practically applied to detect p-NP in a local river and confirmed its validity, showing potential application prospects.

## 1. Introduction

Currently, nitroaromatic compounds are widely used in the chemical industry as raw materials for the manufacture of various pharmaceutical compounds, pesticides, fungicides and dyes. The extensive use of these chemical products results in their wide existence in the environment [1,2]. However, even in trace concentrations, these compounds are harmful to humans, animals and plants and may contaminate freshwater or marine ecosystems. Among them, p-nitrophenol (p-NP) is one of the most common phenolic pollutants, which not only harms our health but causes serious pollution to the environment [3,4]. Therefore, in order to ensure the safety of our living environment, a simple and efficient detection method to detect low concentrations of p-NP, whose concentration may be as low as 0.2 mg/L [5,6], becomes emergent.

Compared with other traditional techniques, electrochemical sensing methods have the advantages of good selectivity, high sensitivity, simple operation, and fast response time [7,8,9]. The detection performance of an electrochemical sensor depends on the chemical properties of the modified materials used in the working electrodes [10,11]. Therefore, the performance of the sensor can be improved by carefully designing the component and structure of the electrode material in terms of selectivity, sensitivity and stability [12,13]. This can be satisfied by designing and developing a new material that can effectively enhance the sensor’s response to p-NP.

Nanomaterials with metal-organic frameworks (MOFs) are currently among the hottest materials studied by scientists in various fields. Because of their high specific surface area, adjustable structure size, low density, and designability, they are all-in-one functional porous polymeric materials [14,15]. Together with great progress for MOFs’ design and preparation, they are widely used in many fields such as gas storage and adsorption, aqueous adsorption and separation, heterogeneous catalysis, targeting theranostics and drug delivery [16,17,18,19,20]. Among the applications in different fields, it is in recent years that MOFs have also attracted the interest of researchers as catalysts in electrochemical reactions [21,22]. It is found that the electrochemical sensing application of MOFs is mainly based on the introduction of catalytically active sites. These sites are introduced through the active metal ions or ligands with electrocatalytic capabilities and further endow MOFs with electrochemical catalytic activity [23]. The metal Cr ion and its oxide, Cr_2_O_3_, have been found to have good catalytic properties and are widely employed in various catalytic fields [24,25]. Herein, based on the excellent properties of MOFs, combined with the excellent catalytic properties of Cr ions, Cr-MOFs were prepared by a simple hydrothermal method [26,27] and used to construct an electrochemical sensor for the quantitative detection of p-NP. The experimental results show that the constructed electrochemical sensor has a good electrochemical response to p-NP and also exhibits good detection ability for p-NP in river water.

## 2. Materials and Methods

Cr(NO_3_)_3_·9H_2_O and H_2_BDC were purchased from Shanghai Aladdin Co. Ltd. (Shanghai, China). Cr-MOF materials were synthesized with the hydrothermal method according to the reported method with modifications [28]. The product was washed by centrifugation, dried in a vacuum, ground and collected for use. An electron microscope (FEI, Hillsboro, America) and XRD apparatus (PANalytical B.V, Almelo, The Netherlands) were employed to characterize the morphology and structure of the product. The electrochemical sensor was fabricated by dropping 10 μL Cr-MOF suspension onto a pre-cleaned GCE (glassy carbon electrode) surface. The details are provided in the Supplementary Information.

## 3. Results and Discussion 

**Figure 1:**
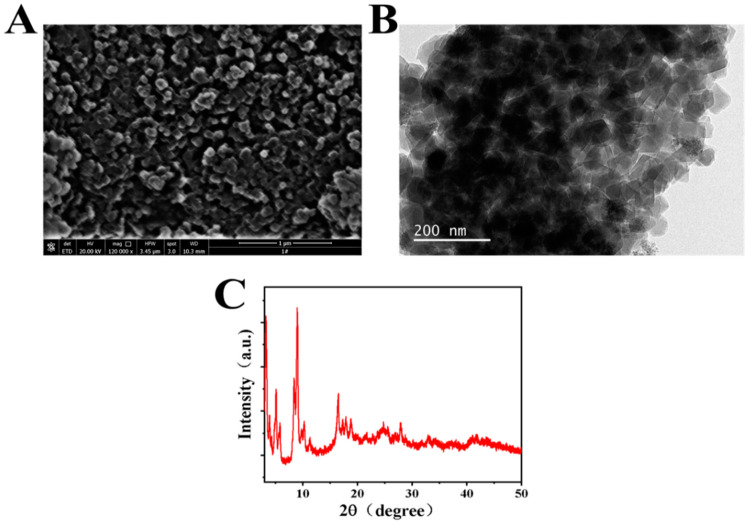
(**A**) SEM, (**B**) TEM, and (**C**) XRD of the prepared Cr-MOF sample.

Figure 1 shows the surface morphology and crystal structure of the prepared Cr-MOF sample. From the SEM image in Figure 1A, it can be seen that the prepared Cr-MOF particles are stacked together in irregular three-dimensional shapes with uniform sizes. Combined with the TEM image in Figure 1B, it can be seen that the particle size of the prepared Cr-MOF particles is about 50 nm with a cubic structure. The crystal structure of the nanoparticles was further characterized by powder XRD, as shown in Figure 1C. The Cr-MOF sample exhibits a series of distinct diffraction peaks located at 3.31°, 5.17°, 5.28°, 9.14°, 10.09°, and 17.1°. These results are well consistent with the previous references about the XRD characterization of Cr-MOF [28,29], which suggest it is a standard cubic structure [30]. This, on the one hand, confirms the successful synthesis of the Cr-MOF nanomaterial; on the other hand, it verifies the good crystallinity of the synthesized nanomaterial.

**Figure 2:**
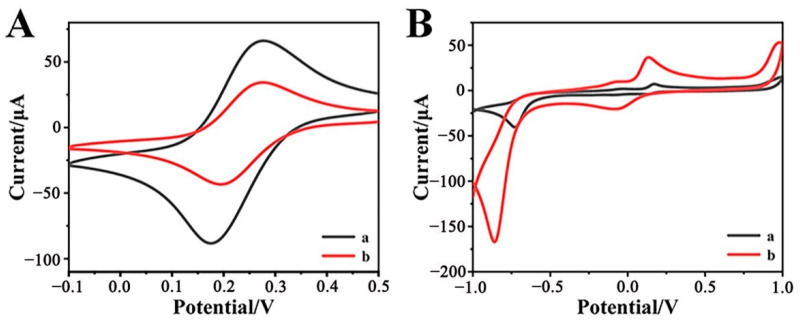
CVs of (a) bare GCE and (b) Cr-MOF/GCE electrodes in the solutions containing (**A**) 0.1 M KCl with 2 mM [Fe(CN)_6_]^3−/4−^ and (**B**) 0.1 M phosphate buffer with 1 mM p-NP at the scan rate of 50 mV s^−1^.

Cyclic voltammetry (CV) technology is commonly applied to investigate the electrochemical properties of modified electrodes. Figure 2A compares the CV curves of bare GCE and Cr-MOF-modified GCE (Cr-MOF/GCE) in the solution containing 2 mM [Fe(CN)_6_]^3−/4−^ electroactive ions and 0.1 M KCl supporting electrolyte. A pair of sharp and well-defined redox peaks on the bare GCE reflects its superior electrical conductivity of the bare GCE; while the apparently weakened redox current on the Cr-MOF/GCE implies that the Cr-MOF modification layer actually increases the electrochemical barrier of the electrode. Figure 2B shows the CV behaviors of the electrodes in phosphate buffer (0.1 M, pH 7.0) containing 1 mM p-NP. The Cr-MOF/GCE exhibits an extremely large reduction current peak that is far superior to the bare GCE. This sudden increase in reduction current is attributed to the electrocatalytic reduction of p-NP by the Cr-MOF nanoparticles at the Cr-MOF/GCE. Based on the outstanding electrocatalytic response of the Cr-MOF nanoparticles to the reduction of p-NP, it is desirable to construct a highly sensitive Cr-MOF/GCE electrochemical sensor for the detection of p-NP.

**Figure 3:**
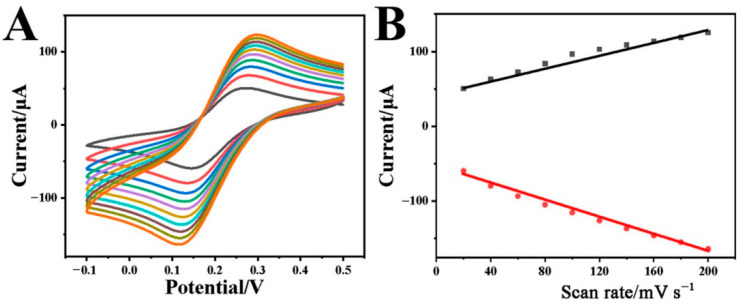
(**A**) CVs of the Cr-MOF/GCE in the solution containing 0.1 M KCl with 2 mM [Fe(CN)_6_]^3−/4−^ at the scan rates varying from 20 to 200 mV s^−1^ and (**B**) the corresponding relationships between anodic/cathode peak current and scan rate.

The electrochemical kinetic property of the Cr-MOF/GCE in the solution containing 0.1 M KCl with 2 mM [Fe(CN)_6_]^3−/4−^ was also studied by CVs at different scan rates. As shown in Figure 3A, both redox current peaks increase with the scan rate rising from 20 to 200 mV·s^−1^. The correlation between peak current (*I*) and scan rate (*v*) was plotted in Figure 3B, presenting the linear proportional relationship.

The anodic peak currents (*I*_pa_) comply with the linear regression Equation (1):

*I*_pa_(μA) = 5.67653 + 0.0557 *v* (mV·s^−1^), R^2^ = 0.9771 (1)

while the cathodic peak currents are in line with the Equation (2):

*I_pc_*(μA) = −4.8442 − 0.04011 *v* (mV·s^−1^), R^2^ = 0.9859 (2)

Both redox reactions suggest a surface-controlled process on the Cr-MOF/GCE.

**Figure 4:**
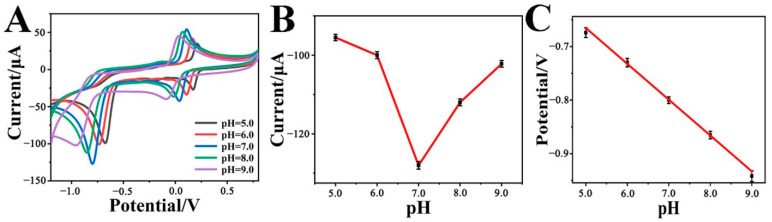
(**A**) CVs, (**B**) reduction peak current versus pH, and (**C**) reduction peak potential versus pH plots of the Cr-MOF/GCE at different pH ranges from 5.0 to 9.0 in 0.1 M phosphate buffer solution with 1 mM p-NP.

In fact, the pH value of the solution has a great impact on the electrocatalytic activity of the Cr-MOF/GCE electrode toward p-NP reduction. Figure 4A shows the CVs of the Cr-MOF/GCE in 0.1 M phosphate buffer solution with 1 mM p-NP under different pH values. It can be seen that both current and potential for p-NP reduction are varied with different pH conditions. The correlation between current and pH was plotted, as shown in Figure 4B. Obviously, the Cr-MOF/GCE exhibits the optimal electrocatalytic capability at pH = 7.0, where the reduction peak current reaches 128 μA. This result suggests that too many or too few protons are unfavorable to the nitro group reduction. Figure 4C reveals the relationship between potential and pH value. The reduction peak potential of p-NP moves negatively as the pH increases from pH = 5.0 to pH = 9.0, in accordance with the linear Equation (3):

*E*_p_(V) = −0.3304 − 0.065 pH, R^2^ = 0.9873 (3)

We refer now to the Nernst Equation (4):

*E*_p_(V) = *E*^θ^ − (0.059 *m*/*n*) pH (4)

where *m* represents the number of protons, and *n* is the number of electrons. The rate of *m*/*n* = 1.1017 is very close to 1, indicating that the p-NP reduction is an equal proton-electron coupled process.

**Figure 5:**
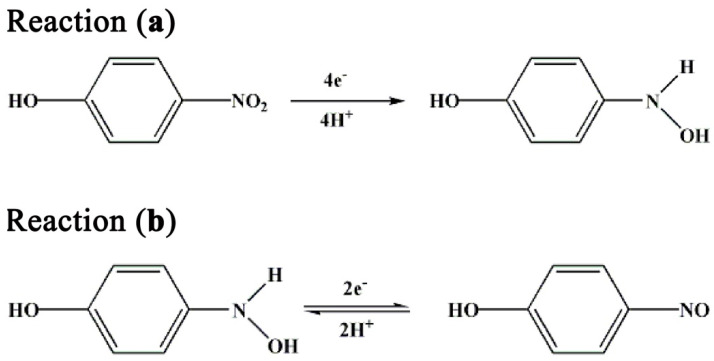
The possible mechanism of p-nitrophenol reduction in this system.

Based on the previous reports about the electrochemical reduction of p-nitrophenol [31,32,33], and combined with the above CV results, the possible mechanism of p-nitrophenol reduction in this system is proposed as follows:

In Figure 5, Reaction (a) is attributed to the reduction that occurred at peak potential ~−0.8 V, which resulted from the four-electron reduction of p-nitrophenol to p-hydroxylaminophenol. Reaction (b), which occurred at redox peak potential ~0.1 V, is a reversible redox reaction resulting from the two-electron redox between p-hydroxylaminophenol and p-nitrosophenol.

**Figure 6:**
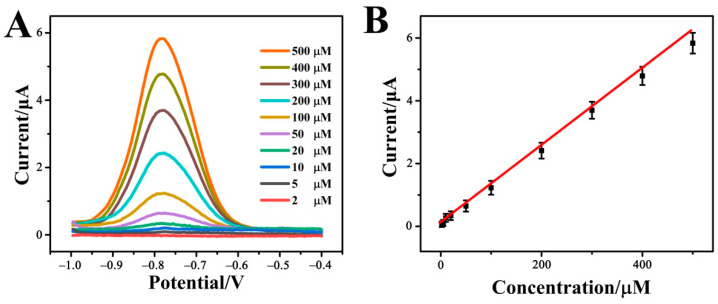
(**A**) DPV response curves and (**B**) linear relationship between DVP peak current and p-NP concentration of the Cr-MOF/GCE electrochemical sensor in 0.1 M phosphate buffer solution (pH = 7.0) containing different concentrations of p-NP from 2 to 500 μM.

The quantitative detection of p-NP by the constructed Cr-MOF-based electrochemical sensor was investigated using the differential pulse voltammetry (DPV) technology. The DPV response curves of different p-NP concentrations in 0.1 M phosphate buffer solution (pH = 7.0) are shown in Figure 6A. As the p-NP concentration gradually increases from 2 μM to 500 μM, the DPV peak current is accordingly increased. The correlation between peak current and p-NP concentration plotted in Figure 6B complies with the linear Equation (5):

*I*_p_(μA) = 0.00749 + 0.00117 *c* (p-NP, μM), R^2^ = 0.9987 (5)

According to the linear relationship, the detection limit of the Cr-MOF/GCE sensor for p-NP is 0.7 μM. This indicates that the Cr-MOF-based electrochemical sensor is highly sensitive to p-NP detection.

**Figure 7:**
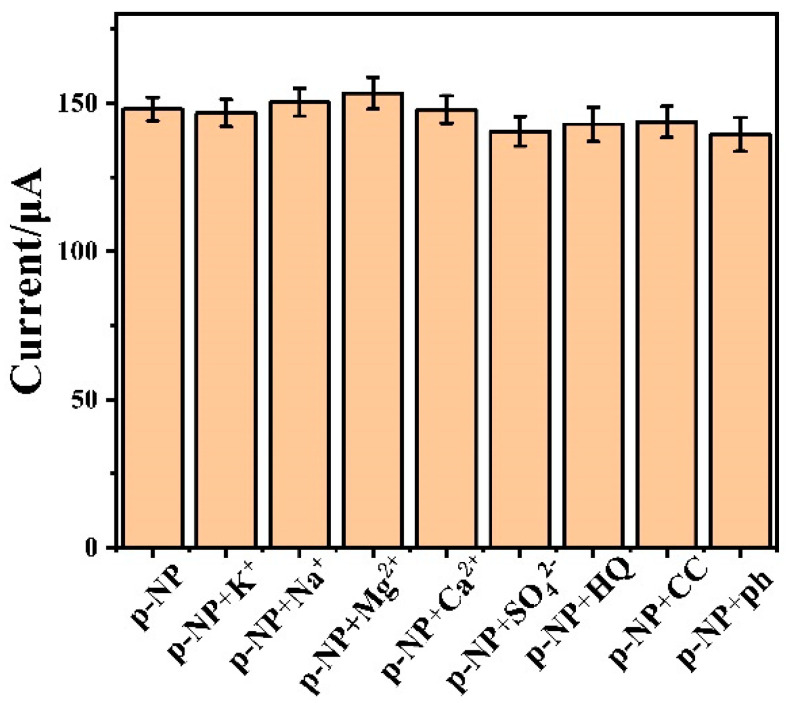
Effect of common interfering substances (0.5 mM) on electrochemical detection of p-NP (1.0 mM) for the Cr-MOF/GCE sensor in 0.1 M phosphate buffer solution (pH = 7.0).

Besides high sensitivity, the anti-interference ability is also very important for a high-performance electrochemical sensor. To investigate the selectivity of the Cr-MOF/GCE sensor to p-NP, a series of common substances in an aqueous solution were added to possibly interfere with the target detection. In this case, magnesium chloride (MgCl_2_), potassium chloride (KCl), calcium chloride (CaCl_2_), sodium chloride (NaCl), sodium sulfate (Na_2_SO_4_), hydroquinone (HQ), phenol (ph) and catechol (CC) were each added with a 0.5 mM concentration. As shown in Figure 7, compared with the p-NP sample, the others with different interference substances present little change in respect of the current response. This indicates that the Cr-MOF/GCE sensor has an exclusively high selectivity to the p-NP response.

To check the reproducibility of the Cr-MOF/GCE sensor, five individuals were constructed and employed for p-NP detection under the same conditions. The detection outcomes reveal that the relative standard deviation (RSD) is only 5.28%, indicating good consistency. To further examine the durability, the Cr-MOF/GCE sensors were kept in a refrigerator for one week and then reused for p-NP detection. The strength of the detection signal could still retain 96.43% of the original value on average, exhibiting high stability.

The Cr-MOF/GCE electrochemical sensor was practically tested by sampling from a local river. Before detection, the insoluble impurities were removed by filtration. Three specimens were measured, and the detection data were listed in Table 1. Actually, no p-NP could be detected in original specimens. After certain concentrations of p-NP were added, the sensor could validly perform quantitative detections with good recovery. This consequence shows that the engineered Cr-MOF/GCE electrochemical sensor has potential competitiveness in practical applications due to its low cost, high sensitivity, and durable stability.

## 4. Conclusions

The Cr-MOF nanoparticles were successfully synthesized using a simple hydrothermal method. Electronic microscopy and powder X-ray diffraction technologies revealed that the nanoparticles with a mean size of ~50 nm present a cubic crystal structure. The electrode reaction on the Cr-MOF-modified GCE is a surface-controlled process, and the Cr-MOF nanoparticles exhibit good catalytic activity toward the electrochemical reduction of p-NP. The constructed Cr-MOF/GCE electrochemical sensor used for p-NP detection shows superior performance, including a low detection limit (0.7 μM), wide linear range (2~500 μM), outstanding anti-interference ability, durable stability, and good recovery in practical detections, and has potential application prospects in water-environmental analysis and monitoring.

## Figures and Tables

**Table 1:** Detection data of p-NP sampled from a local river.

Specimen No.	Original	Added (μM)	Detected (μM)	Recovery	RSD
#1	NBD *	30.0	30.2	100.7%	3.67%
#2	NBD	100.0	101.3	101.3%	2.85%
#3	NBD	200.0	204.6	102.3%	3.19%

* NBD stands for Not Be Detected.

## Data Availability

Not applicable.

## Supplementary Materials

The Supplementary Information containing experimental details can be downloaded at: https://www.mdpi.com/article/10.3390/bios12100813/s1.
