# Supplementary material for: Cr-MOF-Based Electrochemical Sensor for the Detection of P-Nitrophenol
Source: Biosensors (Basel). 2022 Oct 1;12(10):813. doi: 10.3390/bios12100813 (PMC9599216; doi:10.3390/bios12100813)
Supplement: Supplementary file 1 [file biosensors-12-00813-s001.zip › biosensors-1914839-supplementary.pdf]

biosensors

MDPI

Supplementary Information

Cr-MOF-Based Electrochemical Sensor for the Detection of P-nitrophenol

Chao Hu [1,†], Ping Pan [2,†], Haiping Huang [1,*] and Hongtao Liu [2,3,*]

1 School of Chemistry and Chemical Engineering, Jiangxi University of Science and Technology, Ganzhou 341000, China
2 Staff Hospital of Central South University, Central South University, Changsha 410083, China
3 College of Chemistry and Chemical Engineering, Central South University, Changsha 410083, China
* Correspondence: huanghp@jxust.edu.cn (H.H.); liuht@csu.edu.cn (H.L.)
† These authors contributed equally to this work.

1. Experimental

1.1. Materials and Apparatus

$Cr(NO_3)_3{\cdot}9H_2O$ and H_2BDC were purchased from Shanghai Aladdin Co. Ltd. (Shanghai, China). All the other reagents were purchased from Sinopharm Chemical Reagent Co. Ltd (Shanghai, China). Scanning electron microscopy (SEM) images were obtained on an MLA650F FEI (FEI, Hillsboro, OR, USA). Transmission electron microscopy (TEM) images were obtained by FEI Tecnai G20 (FEI, Hillsboro, OR, USA) under 200KV acceleration voltage. The XRD was characterized by a PANalytical X'Pert Pro X-ray diffractometer (PANalytical B.V, Almelo, The Netherlands) with Cu Kα radiation (λ = 0.15418 nm) under 40 kV accelerating voltage and a 40 mA tube current. The scanning speed for XRD was 10 °/min, and the diffraction angle was between 3° and 50°. All electrochemical results, including cyclic voltammetry (CV) and differential pulse voltammetry (DPV), were obtained on a Chenhua electrochemical workstation (CHI 660D, Shanghai, China). The conventional three-electrode system was employed with a modified glassy carbon electrode (GCE) as the working electrode, a saturated calomel electrode (SCE) as the reference electrode, and a Pt wire as the counter electrode.

1.2. Preparation of Cr-MOF

Cr-MOF materials were synthesized with the hydrothermal method according to the reported method with modifications [1]. First, 0.25 g of $Cr(NO)_3{\cdot}9H_2O$ and 0.1 g of H_2BDC were completely dissolved in 10 mL of deionized water, and the mixed solution was sonicated for 30 min. Then, the dispersion was transferred to a 25 mL lined autoclave and hydrothermally reacted in an oven at 180 °C for 15 hours. After the reaction, the kettle was naturally cooled to room temperature, and the product was washed by centrifugation, dried in a vacuum, ground and collected for use.

1.3. Preparation of Modified Electrode

First, the glassy carbon electrode (GCE) was cleaned on a polishing cloth with alumina powder as a suspension. The Cr-MOF suspension with a concentration of 5 mg/mL was obtained by ultrasonication of 5 mL Cr-MOF in 10 mL water for 10 min. The modified electrode was prepared by dropping 10 μL of the suspension onto a pre-cleaned electrode surface. After drying at room temperature, another 2 μL chitosan solution was used to seal.

Reference

1. Duan, F.; Hu, M.; Guo, C.; Song, Y.; Wang, M.; He, L.; Zhang, Z.; Pettinari, R.; Zhou, L. Chromium-based metal-organic framework embedded with cobalt phthalocyanine for the sensitively impedimetric cytosensing of colorectal cancer (CT26) cells and cell imaging, *Chem. Eng. J.* **2020**, *398*, 125452.
